# Automatic Lumen Segmentation in Intravascular Optical Coherence Tomography Images Using Level Set

**DOI:** 10.1155/2017/4710305

**Published:** 2017-02-07

**Authors:** Yihui Cao, Kang Cheng, Xianjing Qin, Qinye Yin, Jianan Li, Rui Zhu, Wei Zhao

**Affiliations:** ^1^The State Key Laboratory of Transient Optics and Photonics, Xi'an Institute of Optics and Precision Mechanics, Chinese Academy of Sciences, Xi'an, Shaanxi 710119, China; ^2^School of the Electronic and Information Engineering, Xi'an Jiaotong University, Xi'an 710049, China; ^3^University of Chinese Academy of Sciences, Beijing 100049, China; ^4^Department of Cardiology, Xijing Hospital, Fourth Military Medical University, Xi'an, Shaanxi 710032, China; ^5^Department of Aerospace Biodynamics, Fourth Military Medical University, Xi'an, Shaanxi 710032, China; ^6^Xidian University, Xi'an, Shaanxi 710071, China

## Abstract

Automatic lumen segmentation from intravascular optical coherence tomography (IVOCT) images is an important and fundamental work for diagnosis and treatment of coronary artery disease. However, it is a very challenging task due to irregular lumen caused by unstable plaque and bifurcation vessel, guide wire shadow, and blood artifacts. To address these problems, this paper presents a novel automatic level set based segmentation algorithm which is very competent for irregular lumen challenge. Before applying the level set model, a narrow image smooth filter is proposed to reduce the effect of artifacts and prevent the leakage of level set meanwhile. Moreover, a divide-and-conquer strategy is proposed to deal with the guide wire shadow. With our proposed method, the influence of irregular lumen, guide wire shadow, and blood artifacts can be appreciably reduced. Finally, the experimental results showed that the proposed method is robust and accurate by evaluating 880 images from 5 different patients and the average DSC value was 98.1% ± 1.1%.

## 1. Introduction

Cardiovascular disease accounts for nearly half of noncommunicable diseases. It remains one of the most leading causes of death in worldwide [1]. It is estimated that about 785,000 Americans each year will get coronary artery disease [2]. Coronary artery disease is mainly caused by the accumulation of atherosclerotic plaques on the coronary artery wall and subsequent decrease of lumen area [3]. As the lumen area decreasing, the blood flow to the heart will be reduced or even the blood vessels become completely blocked. Eventually, it can lead to angina pectoris or sudden death due to the vulnerable plaque ruptures [4]. The lumen area information can be used for diagnosis and treatment of coronary artery diseases, such as evaluating intermediate lesions, selecting balloon or stent dimensions, and deciding the optimal location for coronary stent implantation [5]. Therefore, accurately and quantitatively evaluating the lumen area of coronary artery is an important and fundamental work.

There are mainly two invasive image modalities, intravascular ultrasound (IVUS) and intravascular optical coherence tomography (IVOCT) [6], that can be used to measure the lumen area of coronary artery. IVOCT is based on near-infrared light source rather than acoustic wave source for IVUS imaging. By comparison, IVOCT has much better contrast and spatial resolution (10–20 *μ*m), more than 10 times higher than IVUS [7]. Therefore, based on IVOCT, it can get more accurate measurement of lumen area for the diagnosis and treatment of coronary artery diseases. At present, clinically evaluating lumen area is performed by experts' manual segmentation. However, it is an extremely time-consuming work. Each pullback of IVOCT image sequence generally contains 100–270 effective images. In practice, it will take an experienced analyst more than 4–7 hours to segment all images of a single pullback sequence [7]. In addition, it has problems such as interobserver variability and cannot be reproducible in manual segmentation. Thus, automatic and accurate lumen segmentation method is highly desired.

Several methods for automated IVOCT image segmentation have been reported. Sihan et al. [8] applied Canny filter to detect the edge and then used linking step for segmentation. However, their method may not work well on noisy images due to the fixed threshold. Gurmeric et al. [9] proposed an active contour model based method and also used threshold processing to generate initial contour. The threshold processing suffers from the similar problems on noisy cases. Ughi et al. [10, 11] proposed a lumen segmentation method, which first detected the boundaries on each A-line by several a priori parameters and then smoothed them with a spline fitting. However, some detected points may be far away from the real boundary because the spatial correlation between adjacent A-lines was not incorporated. Tsantis et al. [12] used a Markov random field (MRF) model that iteratively optimized a cost function based on a pixel level estimation for segmentation. Based on pixel level, it may tend to converge to a local regional optimum when it suffers from artifacts noisy. Guha Roy et al. [13] and Wang et al. [14] proposed graph cut models for image segmentation, which also faced the problem of local optimum due to pixel level based evolution. In summary, most of these methods can only be applied on the healthy or nonbifurcation images [15].

**Figure 1:**
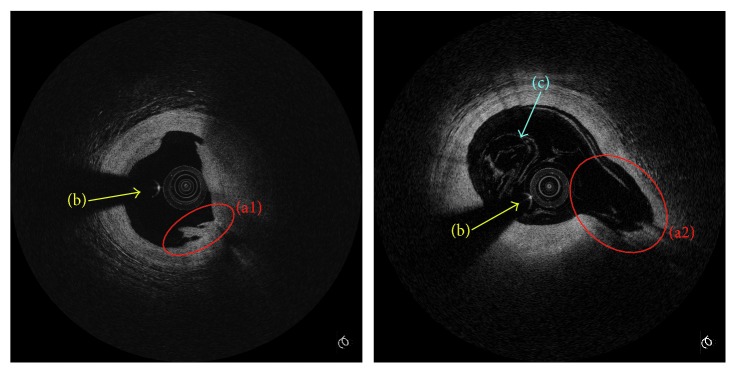
Three challenges of lumen segmentation in IVOCT images. (a) Irregular lumen caused by unstable plaque (a1) and bifurcation vessel (a2). (b) Guide wire shadow. (c) Blood artifacts.

The task of lumen segmentation is difficult due to three challenges. The first is the irregularity and complexity of the lumen boundary. For instance, the lumen with unstable plaque is often irregular [16], as described in region (a1) in Figure 1. Moreover, the shape of artery wall is complex in bifurcation vessel frame, as described in region (a2) in Figure 1. The second challenge is guide wire shadow. The guide wire is used to guide catheter through the coronary artery but its shadow makes the contour incomplete, as the arrows (b) referred in Figure 1. Thirdly, luminal blood artifacts commonly exist in the lumen of IVOCT images, as the arrow (c) refers in Figure 1, which brings a great challenge to distinguish the real boundary from the edges of artifacts.

In kinds of medical image segmentations methods [17–19], level set based method [20–22] is the most promising one to delineate local irregular shape as well as incorporate the global image information. However, directly using the classical level set based method is not competent due to the specific challenges as described above. In this paper, a divide-and-conquer strategy is firstly proposed to address the challenge of guide wire. In this strategy, the region shadowed by the guide wire is removed first. Then, the contour of the region without shadow is detected. Finally, the complete contour is reconstructed from the detected contour points by using polynomial fitting. To reduce the effect of artifacts, a narrow image smooth filter is proposed which prevent the leakage problem of level set meanwhile. According to our best knowledge, this is the first work to use level set for automated IVOCT image segmentation. Compared with the state-of-the-art method [10], it is shown that the proposed method gets more accurate and more robust results.

The rest of this paper is organized as follows. The proposed level set based segmentation method and its workflow are presented in Section 2.  Section 3 gives the experiment results and performance of the proposed method. The conclusions are drawn in Section 4.

## 2. Method

In this section, we first introduce the gradient-based level set model which is used to address the irregular lumen challenge. Then, a proposed divide-and-conquer strategy is described against guide wire challenge in second subsection. In third subsection, a narrow image smooth filter is presented against the artifacts challenge. Finally, the workflow of this method is given in the last subsection.

### 2.1. Level Set Model against Irregular Lumen Challenge

Level set based segmentation method can be roughly classified as region-based model [23, 24] and gradient-based model [25, 26]. Region-based level set evolves by the force of minimizing the intensity inhomogeneity of each region, which assumes the image intensity is relatively homogeneous. This model is based on a global energy function. However, if a region contains complicated intensities, it will tend to result in either oversegmentation or undersegmentation. In IVOCT images, the guide wire shallow and blood artifacts lead to the intensity inhomogeneity of intravascular lumen. Thus, it is unsuitable to use the region-based segmentation model.

Gradient-based model mainly utilizes edge information for segmentation, which can usually obtain better result for the target boundary with large gradient magnitude. This model does not need to assume image intensities as homogeneity and thus has a wider application area. For IVOCT image, the lumen boundary is usually obvious and the gradient magnitude is large. Therefore, gradient-based level set method can be used for IVOCT image segmentation.

Distance regularized level set (DRLS) model is a gradient-based level set framework presented by Li et al. [26] which is a more stable model. It improves the efficiency and effect of segmentation by avoiding the reinitialization problem in traditional level set methods [27, 28]. The specific energy function of DRLS model is

(1) $$\begin{array}{c} E_{drls}\left(\;\phi \;\right)=\mu R\left(\;\phi \;\right)+\lambda L\left(\;\phi \;\right)+\alpha A\left(\;\phi \;\right), \end{array}$$

where *μ* > 0, *λ* > 0, and *α* ∈ *ℜ* are the corresponding parameters and the function *ϕ* is a level set function. The first term is a level set regularization term

(2) $$\begin{array}{c} R\left(\;\phi \;\right)=\int_{\Omega }p\left(\;\left|\;\nabla \phi \;\right|\;\right)dx, \end{array}$$

where *p* is a double-well potential [26] with two minimum points at 0 and 1. During evolution, it forces the gradient magnitude of level set approach to one of its minimum points. This can maintain a signed distance property near the zero level set to keep the level set contour stable. By this way, the regularization term can avoid the reinitialization problem in traditional level set methods and maintain contour smooth during evolution. Thus, this term is also called the internal energy.

The second term is length term

(3) $$\begin{array}{c} L\left(\;\phi \;\right)=\int_{\Omega }I_{g}\delta \left(\;\phi \;\right)\left|\;\nabla \phi \;\right|dx, \end{array}$$

where *I*_*g*_ is an edge indicator function defined as *I*_*g*_(*x*) = 1/(1 + |∇*I*(*x*)|^2^). The length term is a line integral of *I*_*g*_ along the contour of zero level set. When the zero level set arrives at target boundary, *L*(*ϕ*) is minimized. The aim of this term is to attract the zero level set to the edge of image.

The third term is an area term

(4) $$\begin{array}{c} A\left(\;\phi \;\right)=\int_{\Omega }I_{g}H\left(\;\phi \;\right)dx, \end{array}$$

where *H* denotes Heaviside function as

(5) $$\begin{array}{c} H\left(\;f\left(\;x\;\right)\;\right)=\left\{\begin{array}{cc} 1, & f\left(x\right)\geq 0,\\ 0, & f\left(x\right)<0. \end{array}\right. \end{array}$$

The area term computes the weighted area of *Ω*_*ϕ*_^+^ = {*x* : *ϕ*(*x*) > 0}. It equals the area of *Ω*_*ϕ*_^+^ when *I*_*g*_ = 1. The aim of the area term is to speed up level set evolution. Particularly when the initial contour is far away from the target boundary in case of bifurcation, this term is very important. Let *ϕ* be inner positive and outer negative; then if the initial contour is outside the target, *α* is set as *α* > 0 so the zero level set can shrink to the target boundary; if the initial contour is inside the target, *α* is set as *α* < 0 so the zero level set can expand to the target boundary. When the zero level set arrives at the target boundary, *I*_*g*_ is very small to slow down the speed of shrink or expand.

The corresponding level set evolution equation can be obtained by minimizing the energy function (1)

(6) ∂ϕ∂t=μ divd∇ϕ∇ϕ+λδϕdivIg∇ϕ∇ϕ−αIgδϕ,

where *d*(|∇*ϕ*|) is a diffusion rate function. Using this model, the initialization of level set function can be simplified as a binary step function by setting the inner zero level set as 2 and the outer as −2. While the zero level set evolves in a flat region with small image gradient |∇*I*(*x*)|, the edge indicator function *I*_*g*_(*x*) approximately equals 1. So the level set variation ∂*ϕ*/∂*t* is large to make the evolution move forward. While the zero level set arrives at the sharp edge, the image gradient |∇*I*(*x*)| is very large and *I*_*g*_(*x*) is close to zero. So the energy function is minimized and the evolution stops. For more details about the DRLS model, refer to [26].

### 2.2. Divide-and-Conquer Strategy against Guide Wire Challenge

However, if the DRLS model is directly used for IVOCT image segmentation, there is an obvious occlusion problem resulting from the guide wire. It may lead to stopping at the top of guide wire or a serious leakage of zero level set. Moreover, the edges nearby the region of guide wire also have large gradient which will mislead the segmentation. In this paper, a divide-and-conquer strategy is proposed to address the effect of guide wire.

**Figure 2:**
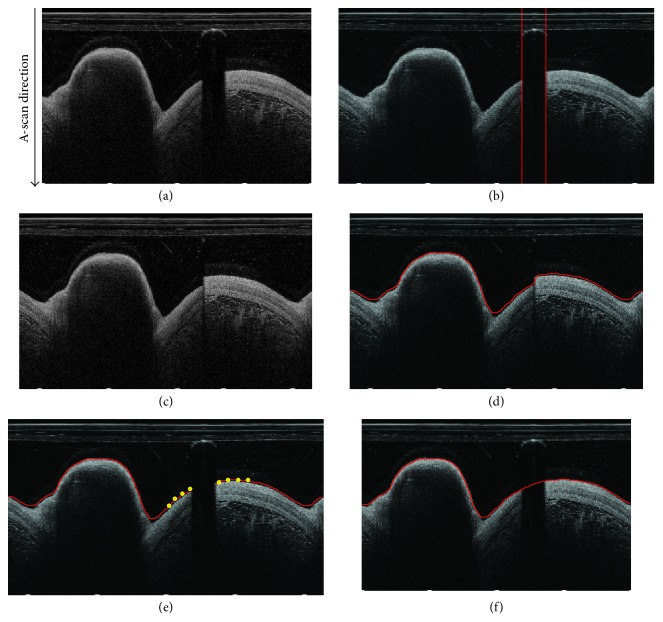
(a) Polar coordinate. (b) Separated guide wire region. (c) Merged image. (d) Segmentation of the region with guide wire removed. (e) Selected 2*K* points for fitting. (f) Complete contour.

Generally, for computational convenience, the IVOCT images are first transformed from Cartesian space coordinate into Polar coordinate by Hough transform [29], as shown in Figure 2(a). In Polar coordinate, the vertical axis is called A-scan direction.

Firstly, assume that the region of guide wire is calculated, it can be separated by two parallel lines, as shown in Figure 2(b). Then the region of guide wire can be easily removed by merging the regions outside of the two separated lines, as shown in Figure 2(c). After the guide wire is removed from the image, the contour is consecutive. Then, the level set model is competent to segment this consecutive boundary. After implementing the level set, the image of Figure 2(c) can be segmented as shown in Figure 2(d).

When it gets the segmentation of parts of the boundary without guide wire, the complete contour can be computed by polynomial fitting. Specifically, it first gets *K* points from both sides of the guide wire, as the yellow points denoted in Figure 2(e). Based on these 2*K* points, the boundary in the region of guide wire can be fitted by *M*-order polynomial fitting. Finally, the complete contour is composed of the level set segmentation result and the fitting result, as shown in Figure 2(f).

**Figure 3:**
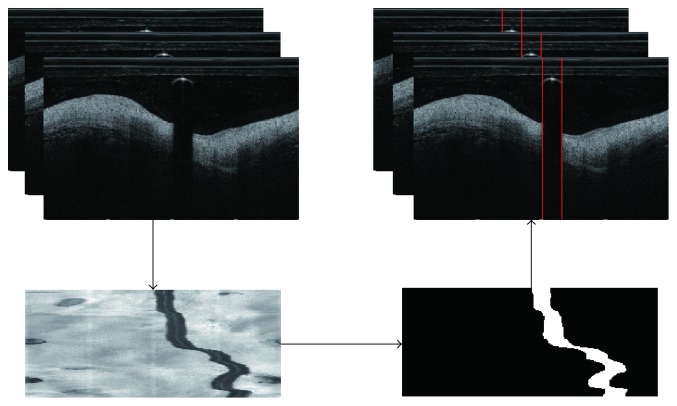
The work follow of guide wire segmentation.

The above algorithm is based on the assumption that the guide wire region was calculated. Thus, the next important thing is to calculate the guide wire region automatically. It can be seen that an obvious feature of guide wire is that it has a bright reflection and followed by a dark shadow immediately. Another important feature is its spatial continuity along the pullback direction [11]. Thus, the guide wire can be segmented by utilizing these two features as follows. Firstly, accumulate the value of pixels on each column and then normalize the summation so that each image can be mapped as a single stripe. Then, along the pullback direction, stacking the stripes mapped by images, it can get a mapped image, as shown in lower left of Figure 3. In this image, each row is corresponding to an single frame image. It can be seen that the guide wires formed an obvious dark and river-liked region. Thus, it is relatively easier to segment in the mapped image than in the original image. By using the classical Otsu's method [30] and followed by morphological closing and area constraint and dilation, the guide wire regions can be segmented, as shown in lower right of Figure 3. Finally, each image can be segmented by computing the boundary points of the corresponding stripe on the segmented mapped image, as shown in upper right of Figure 3.

### 2.3. Narrow Image Smooth Filter against Artifacts Challenge

**Figure 4:**
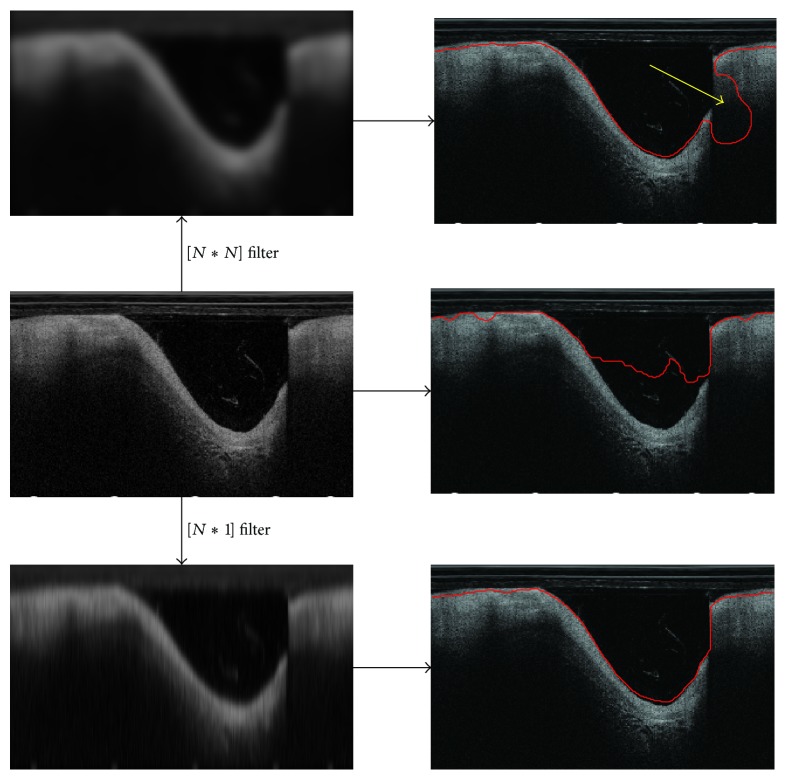
Comparison of different image smoothing filter and their corresponding segmentation results.

However, when facing the heavy noise of blood artifacts, it may still be failed. Take an image, for example, as shown on the left side of the middle of Figure 4; there are some artifacts noise in the lumen. If the level set model is implemented directly for segmentation, the edges of artifacts will prevent the evolution of the zero level set. The final segmentation will be failed, as shown on the right side of the middle of Figure 4.

Generally, a smooth filter can be taken to reduce the noise effect. For example, if the image on the left side of the middle of Figure 4 is processed by a Gaussian filter, as shown on the left side of the top of Figure 4, the boundary of artifacts is smoothed, and it is no longer preventing the evolution. Based on the smoothed image, the segmentation result is shown on the right side of the top of Figure 4. Compared with the direct use of segmentation, the smoothed segmentation result is no longer affected by the artifacts. But it should be noted that leakage problem occurs in the region of the yellow arrow. These regions have a common feature that the boundary changed dramatically along A-scan direction, which usually occurred at the region with guide wire removed or at the region of bifurcation vessel. The boundary in this region will be blurred by common smooth filter.

To overcome this problem, we proposed a narrow Gaussian kernel for image smoothing. The artifacts and catheter are mostly perpendicular to the A-scan direction and narrow relative to the lumen boundary. Thus, a large Gaussian kernel filter can smooth the noises of artifacts and catheter. While if the kernel of filter is narrowed enough, the real lumen boundaries which changed dramatically along A-scan direction will not be affected. As shown in the right side of the bottom row of Figure 4, the segmentation is satisfactory. The top row is based on a *N* × *N* sized Gaussian kernel, while the kernel size of bottom is *N* × 1, both of which are based on the same deviation parameter *σ*.

### 2.4. Overall Workflow

**Figure 5:**
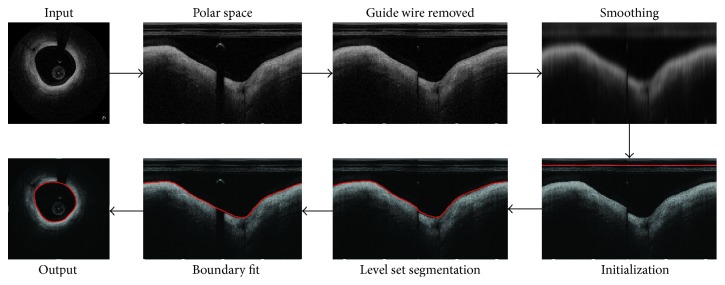
The overall workflow of the proposed method.

The segmentation framework is shown in Figure 5. For the segmentation of each frame, it is first transformed into Polar coordinate space for computational convenience. Thus, the zero level set is initialized as a straight line and the region above the line is used as the region *Ω*_*ϕ*_^+^ for the computation of the level set function *ϕ*(*x*). More specifically, the details of the workflow in Figure 5 can be summarized as 6 steps as follows:

1. Coordinate transform: each image to be segmented is first transformed from the Cartesian coordinate space to the Polar coordinate space by Hough transform [29].
2. Guide wire remove: the guide wire region can be located by the segmented stacked sequence image and then it can be removed, as described in the second paragraph of Section 2.2.
3. Smoothing: based on the image with guide wire removed, the proposed narrow Gaussian filter is implemented to reduce the artifacts and prevent leakage problem meanwhile, as described in Section 2.3.
4. Level set segmentation: first, the initialization of zero level set is set to be a straight line above the diameter line of catheter to make sure the initialization is inside the lumen, as shown by the red line in the left bottom of Figure 5. Then, the DRLS is implemented to detect the lumen boundary. It should be noted that the evolution of level set is always implemented on the preprocessed image, but to intuitively illustrate the contour of initialization and segmentation result, we showed them on the source image instead, as shown in Figure 5.
5. Boundary fit: after the lumen boundary of the region with guide wire removed is segmented, the complete contour can be computed from the segmented boundary, as described in detail in the third paragraph of Section 2.2.
6. Coordinate inverse transform: finally, with the inverse transform, which is from the Polar coordinate space to Cartesian coordinate space, the input image can be segmented as shown in lower left of Figure 5.

## 3. Experiments

### 3.1. Materials, Evaluation, and Parameter Settings

The proposed method was tested on 880 IVOCT images from 5 patients. Each pullback contains effective frames varying between 117 and 271 for different patients. All the IVOCT pullbacks were obtained by using the FD-OCT system (C7-XR system, St. Jude, St. Paul, Minnesota) and the Dragonfly catheter (St. Jude). The data were acquired with the following parameters of pullback speed: 20 mm/s, frame rate: 100 frames, axial resolution: ≤20 *μ*m, and lateral resolution: 25–60 *μ*m.

For the quantitative evaluation, Dice similarity coefficient (DSC) was employed as the metric. Let *S*_*a*_ denote the shape of automatic segmentation and *S*_*m*_ denote the shape of manual segmentation; the overlap area between *S*_*a*_ and *S*_*m*_ is defined as

(7) $$\begin{array}{c} DSC\left(\;S_{a},S_{m}\;\right)=\frac{2\left|S_{a}\cap S_{m}\right|}{\left|S_{a}\right|+\left|S_{m}\right|}\times 100\%. \end{array}$$

In our experiments, we empirically fixed the insensitive parameters *μ* = 0.2 and *λ* = 5 in level set model. The parameter *α* in (1) controls the evolution speed of level set. Due to the complicated intensities around catheter, the initial value of *α* should be large to speed up the level set out of the catheter area. Then, near the boundary of lumen, the value of *α* should be small to avoid leakage. Thus, the parameter *α* was set as *α* = 9 in the first 100 evolutions of level set and *α* = 2 in the rest of evolution. In the divide-and-conquer strategy, the parameters *K* and *M* are set to 10 and 9 by experiments. In the narrow image smooth filter, *N* is set to be 20 according to the width of lumen boundary in this work, and *σ* was set to be 4. The parameters were tuned and the best values are chosen. Then they are fixed for the 5 sequences. In our tests, no significant variation of segmentation result was obtained when varying the parameters around the given values.

### 3.2. Performance of the Proposed Method

#### 3.2.1. Performance of Divide-and-Conquer Strategy

**Figure 6:**
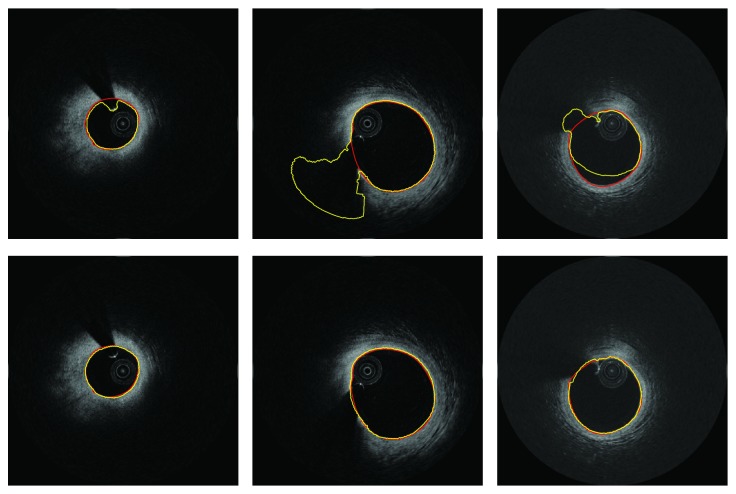
Examples show the performance of the divide-and-conquer strategy against guide wire challenge. First row: direct segmentation. Second row: results based on the proposed divide-and-conquer strategy. Red: ground truth. Yellow: automatic segmentation.

As shown in Figure 6, we compared the segmentation results of directly using level set (first row) and with the proposed divide-and-conquer strategy (second row). It can be seen that without guide wire removed, the results of direct segmentation may have two problems. The first is the zero level set may be prevented by the guide wire, as shown in left top of Figure 6. This problem usually occurred in the cases that the guide wire is close to the lumen boundary. The second problem is that the results may suffer from heavy leakage in guide wire region, as the case in the middle top of Figure 6. The leakage problem results from the region where there is no significant boundary on the shallow of guide wire. In some cases, like the image of right top of Figure 6, the result may be suffered from both problems, while, based on the proposed strategy, the boundary in guide wire region can be fitted accurately, as shown in the third row in Figure 6.

#### 3.2.2. Performance of Narrow Image Smooth Filter

**Figure 7:**
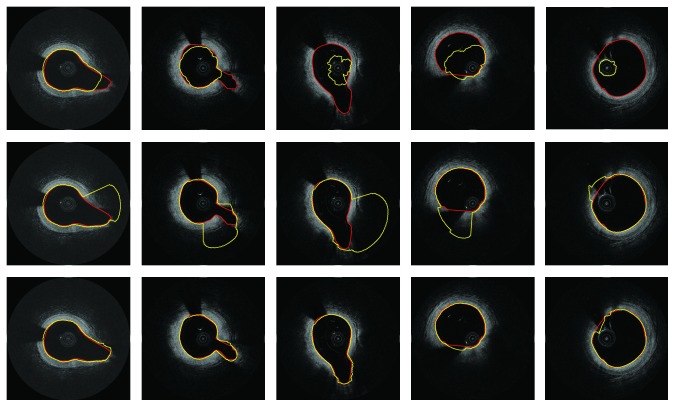
Examples compare the performance of different image smoothing methods. First row: without smoothing. Second row: smoothing by *N* × *N* kernel. Third row: smoothing by the proposed method. Red: ground truth. Yellow: automatic segmentation.

To illustrate the performance of the proposed image smoothing method, it is compared with the results with no smoothing, with smoothing by *N* × *N* kernel sized filter and with the proposed narrow image smooth filter. As shown in Figure 7, the results of first row are without smoothing. It can be seen that the evolution of level sets cannot reach to the real boundary due to the prevention of blood artifacts. In second row, the results are based on *N* × *N* kernel smoothing; the level set can pass through the artifacts. However, heavy leakage problem occurs in week boundary region, especially in bifurcation region. On the contrary, based on the proposed narrow image smooth filter, the robust segmentation results can be obtained, as shown on the second row of Figure 7.

### 3.3. Comparison with Other Methods

#### 3.3.1. Comparison Method

To further validate the performance of our proposed method, we tested the same images by method [10]. In their method, it removed guide wire region firstly. Then, it searches the edge point based on each single A-line by some thresholds. Finally, these searched points are fit to result in a segmentation.

#### 3.3.2. Qualitative Comparison

**Figure 8:**
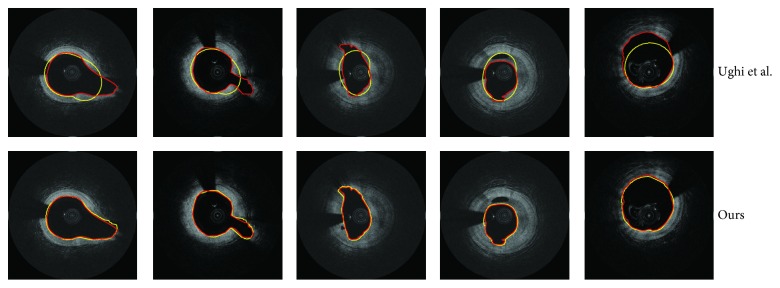
Examples show the segmentation results of five frames based on the two methods. Red: ground truth. Yellow: automatic segmentation.

Some segmentation results based on the two methods are shown in Figure 8. It can be seen that the results of our proposed method are the most approximate to the ground truth. In the first two columns, the method [10] cannot be competent to segment the bifurcation vessel. In the bifurcation region, the boundary is not significant, and the thresholds in search step may fail on the noise. Thus, it will affect the subsequent fitting results. In the next two columns, compared with the results of method [10], our method is more able to fit the irregular lumen of unstable plaque. Comparing the results of the last column, it can be seen that the method [10] is more sensitive to the noise of blood artifacts and catheter than ours.

#### 3.3.3. Quantitative Comparison

**Figure 9:**
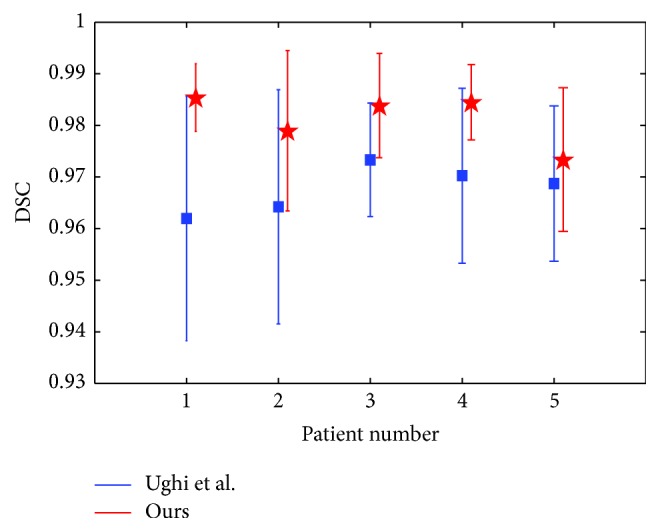
The average DSC of segmentation results based on the two methods.

The quantitative evaluation results of the two methods are shown in Figure 9. It can be seen that the results of our method are significantly better (*p* < 0.05, *t*-test) than the results of Ughi et al. [10] in most cases. For the overall comparison of the two methods, the average DSC values were computed by averaging the segmentation results over all the patients. With the whole test images, the average DSC value of the method of Ughi et al. [10] is 96.9% ± 1.8%, while the DSC of the proposed method is 98.1% ± 1.1%, with an improvement of 63.2%.

### 3.4. Results for Clinical Analysis

**Figure 10:**
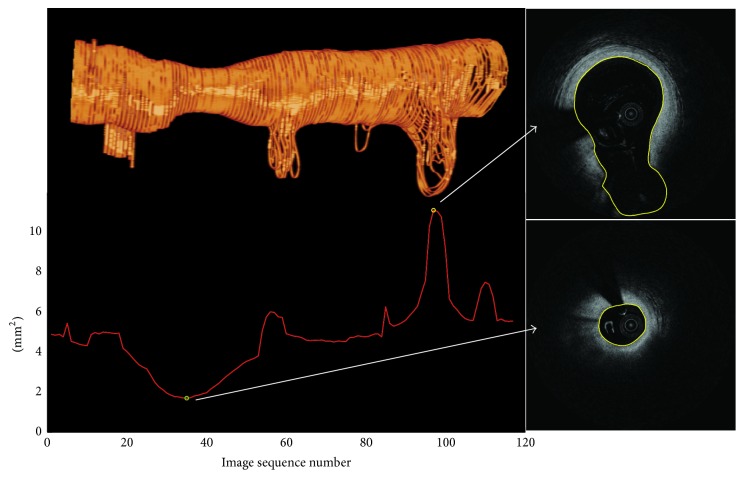
The whole vessel can be 3D rendered with the automatically segmented lumen contours. Red curve: lumen area curve. Green circle: the location of minimal luminal area. Yellow circle: the location of the biggest luminal area.

Based on segmentation results, the 3D structure of coronary artery lumen can be reconstructed for cardiologists insight. Some quantitative parameters can be automatically computed for diagnosis and treatment. For example, the minimal luminal area (MLA) reflects the degree of coronary narrowing and the lumen area curve helps the cardiologists selecting the balloon and stent and evaluating the optimal location for implantation of a coronary stent.  Figure 10 shows a 3D reconstruction result based on VTK [31], which can provide the 3D structure of coronary artery lumen intuitively for cardiologists. Its lumen area curve is plotted by a red curve as shown in Figure 10. The lumen area is computed by accumulating the pixel number and multiplying by the spacing of each pixel. Thus, the MLA and its location can be easily obtained from the curve. In this case, the location of MLA is located at the green circle shown in Figure 10. Its corresponding frame and the segmentation of MLA are shown in the right bottom in Figure 10. Moreover, the biggest luminal area is located by a yellow circle, and its corresponding frame is shown in the right top of Figure 10. It can be seen that it contains a big bifurcation vessel.

## 4. Conclusion

In this paper, a novel level set based lumen segmentation method is proposed for IVOCT images. The main contributions of this work are included as follows: (1) To the best of our knowledge, this is the first work introducing level set model to segment artery lumen contour in IVOCT images. (2) By using a divide-and-conquer strategy, the challenge of guide wire shadow is addressed. (3) By analyzing the direction features between the artifacts and lumen contour, a special narrow image smooth filter is proposed to reduce the blood artifacts. Finally, by comparing with a state-of-the-art method [10], the experimental results show the proposed method is robust and promising. In future work, shape prior base method may be applied to constrain the evolution of level set to get more accurate results.
